# Bentonite Clay as a Natural Remedy: A Brief Review

**Published:** 2017-09

**Authors:** Maryam MOOSAVI

**Affiliations:** 1. Dept. of Physiology, School of Medical, Shiraz University of Medical Sciences, Shiraz, Iran; 2. Nanomedicine and Nanobiology Research Center, Shiraz University of Medical Sciences, Shiraz, Iran

**Keywords:** Bentonite clay, Medicine, Health, Remedy

## Abstract

**Background:**

From old times, the human kind has used clays, externally or internally, for maintaining body health or treating some diseases. Meanwhile there are few scientific articles reviewing the beneficial effects of clays on body function. Bentonite clay is one of the available clays in nature, used as traditional habits, and remedies in many cultures.

**Methods:**

These articles explored among 2500 scientific articles published in PubMed to sort the scientific works have been done on the effects of this clay on body function (it was about 100 articles).

**Results:**

Bentonite has a broad range of action on different parts of body.

**Conclusion:**

As traditional remedies seem to have a deep root in maintaining body health, it merits doing more research works on bentonite clay and its impacts on body function.

## Introduction

Geophagy has been considered as an adaptive behavior in humans and animals (1) and the clays have been considered the healing materials from ancient times. As geophagia is an occasionally habits of animals and humans (babies and pregnant women), physiologically it is assumed that earth might have some beneficial effects on body function. Bentonite is absorbent aluminium phyllosilicate clay. It is named after Fort Benton, Wyoming where its largest sources are found. Its other name, Montmorillonite clay, stems from the region of France called Montmorillon, where it was first found. It has been used and eaten from ancient time till now as human believes in its therapeutic benefits. When it is mixed with water a paste is formed which has been used both externally and internally. In some places such as Iran, it has been widely used as a hair cleanser from old time. There is enough literature showing its safety after chronic oral consumption (2–6). As this clay is abundant, non-expensive and natural, this article aimed to review the scientific papers reporting the effects of this clay on body.

## Methods

The articles were searched in Medline between the English language journals. These articles had no time limit and the data were extracted from the first article in this field. The keyword included mainly bentonite to assess all the articles in medicine field. The inclusion criteria was a paper reporting any effect of bentonite on health in any animal and the exclusion criteria was the other information not in medicine field.

### The effect of bentonite on body function Detoxification

Bentonite clay has been shown to act as a detoxifying agent. This property is referred to its poly-cationic nature, which leads to absorption of negative charge toxins (7).

T-2 is a trichothecene mycotoxin which is a naturally occurring mold byproduct of Fusarium fungus and is toxic to humans and animals. When rats ingested bentonite for 2 weeks before T-2 toxicosis, a significant increase in fecal excretion of this toxin and a decrement of that in muscle has been reported (8) which indicates the healing effect of bentonite against T2 toxicosis.

Aflatoxins are poisonous and cancer-causing chemicals that are produced by certain molds which primarily affects liver. Firstly in 1989 Dvorak et al reported that bentonite is able to reduce water aflatoxin to 66% of its primary concentration which showed the adsorbing capacity of bentonite for aflatoxin (9). Schell et al showed that in pigs, when clay is added to aflatoxin-contaminated corn, it partially restored liver function without greatly affecting mineral metabolism (10). In rabbits fed with aflatoxin-contaminated diet, there is a deficiency in reproductive function and adding bentonite to the aflatoxin-contaminated diet improved the physical semen characteristics of buck and reproductive performance traits of doe rabbits (11). There are numerous animal feeding studies, which demonstrated that bentonites, can bind aflatoxins in ingested feed and reduce or eliminate the toxicity (12–15). Bentonite reduces the bioavailability of aflatoxin (16) and decreases the amount of Aflatoxin M1, which is a hydroxylated metabolite of aflatoxin B1 in Ghanaian population (17). Currently the safety of dietary bentonite is shown in different studies including animals and humans (2, 4–6). Besides, it does not affect the serum concentrations of important vitamins and nutrient minerals in humans (3). Then bentonite is being used in humans as a dietary intervention to prevent and cure aflatoxin toxicity.

Organochlorine pesticides are known to be one of the most persistent organic pollutants present in the environment. They are highly toxic, chemically and biologically stable and have the tendency to accumulate in organisms. Bentonite is reported to have the capacity to absorb pesticides due to its cationic nature (18). It has the potential for sorption the pesticide endrin possibly due to a combination of hydrophobic and charge-dipole interactions (19) in lab conditions. Paraquat is an herbicide, which is highly toxic to mammals, including human. Following ingestion of paraquat in high dose, hepatic, cardiac or renal failure or death might occur. In smaller doses the symptoms like as respiratory distress, renal dysfunction or, occasionally, jaundice or adrenal cortical necrosis appear. Gastric lavage with bentonite removes paraquet. It might acts as an adsorbent to reduce the effect of this toxin on body (20, 21). Then it might be concluded that bentonite counteract pesticides and herbicides toxicity.

Some toxins might exist in foods of livestock. Lantana camara, a species of flowering plant, is known to be toxic to livestock such as cattle, sheep, horses, dogs and goats. When cows which were poisoned with Lantana camara, were treated 5 days after with oral bentonite, five of 6 calves given bentonite recovered while 5 of 6 calves in the control group died and comparing with activated charcoal, the plasma total bilirubin concentration was statistically more appropriate in bentonite treated cows (22). Then bentonite is suggested to act as a cheaper and more effective treatment comparing with charcol.

Metal toxicity or metal poisoning is the toxic effect of certain metals in certain forms and doses on life. Lead poisoning is a medical condition in humans and other vertebrates caused by increased levels of the heavy metal lead in the body. Lead interferes with a variety of body processes and is toxic to many organs and tissues including the heart, bones, intestines, kidneys, and reproductive and nervous systems. In pigs, the feeding supplementation of montmorillonite for 100 days, reduced lead concentration in blood, brain, liver, bone, kidney and hair (23). Copper toxicity, also called copperiedus, refers to the consequences of an excess of copper in the body. Consistently in sheep, it was concluded the dietary Cu bioavailability could be decreased by oral supplements of bentonite (24). Cadmium is an extremely toxic metal commonly found in industrial workplaces. The exposure of carp (*Carassius auratus* to dietary cadmium caused oxidative stress, while montmorillonite supplemented in diet reversed relatively cadmium-induced oxidative damage in liver and kidney (25). Bentonite is additionally reported to decrease cadmium induced cytotoxicity and genotoxicity in Nile tilapia fish (26). Generally, it seems that bentonite is a reliable treatment for metal poisoning.

Generally, it seems that bentonite can be considered as a treatment in different types of toxicities although in some sorts of toxicities such as locoweed toxicosis in rats, it is not effective in alleviating the symptoms (27).

### Skin and hair

For a long time bentonite has been used externally on skin. Poison ivy and poison oak are the most common causes of allergic contact dermatitis in North America. Quaternium-18 bentonite lotion has been shown to act effectively in preventing or diminishing experimentally produced poison ivy and poison oak allergic contact dermatitis (28, 29). Irritant and allergic hand dermatitis is considered as a difficult problem to be controlled in individuals who are unable to avoid causative exposures. Using Quaternium-18-Bentonite in a moisturizing cream has been shown to improve chronic hand dermatitis in a majority of individuals with previously uncontrolled dermatitis (30). In diaper dermatitis, which is one of the most common skin disorders of infancy, bentonite is reported to act better and faster than calendula, the current treatment of this type of dermatitis (31, 32).

Bentonite could act as a barrier for toxic organophosphorous compound transfer across the skin, which suggests its physical protective action on skin (33). In case of sunscreens, it has been reported that inclusion complexes of commercial sunscreens in montmorillonites have optimized functional properties such as water resistance and skin adherence, which make them good substrate in these types of skin product (34). Furthermore, it is reported that sun lotions containing specific proportion of bentonite mineral are more potent than commercially available sun lotion in absorbing the highest level of UV light (35).

Bentonite is also shown to act efficiently in healing of skin lesions and ulcers (36).

Although in many areas such as Iran it was long used as a hair cleaner and softner, there is not any scientific article assessing its effect on hair. Meanwhile it is shown that in sheep bentonite increases wool growth (37).

### Gastrointestinal tract

For a long bentonite was considered as a treatment of diarrhea. At 1961, it was shown that orally administered bentonite treat 97% of cases with different causative factors of diarrhea (virus infection, food allergy, spastic colitis, mucous colitis, and food poisoning) (38).

Irritable bowel syndrome (IBS) is a common, long-term condition of the digestive system. It can cause bouts of stomach cramps, bloating, diarrhoea and/or constipation. When bentonite (3 g, t.d. for 8 weeks) was administered in patients with irritable bowel syndrome (IBS) it affected this syndrome. Although pain or discomfort was not significantly improved in the entire IBS population treated with montmorillonite in comparison with placebo, it modulates bowl habits in constipation-predominant IBS (39). The montmorillonite which was combined with zinc in diets, improved growth, alleviated post weaning diarrhea, and enhanced intestinal mucosal integrity and the digestive enzyme activities in pancreas and small intestinal contents of pigs (40).

While bentonite can absorb many organic and inorganic materials in GI tract, it is reported not to affect mineral metabolism (10) and absorption (41). Gut flora is the complex community of microorganisms that live in the digestive tracts of humans and other animals. These microorganisms benefit the host by fermenting dietary fiber into short-chain fatty acids and synthesize vitamin B and vitamin K as well as metabolizing bile acids, sterols, and xenobiotics (42). Intragastric administration of bentonite to rats for 28 days leads to hyperproduction of colonic yeast micro-flora (43). Then bentonite might help nutrients absorption through increasing gut flora activity.

### Kidney

Creatinine is a breakdown product of creatine phosphate in muscle and its serum content is an important indicator of renal health as it is excreted by kidneys. Creatinine is able to diffuse from the blood vessel to the intestine and be reabsorbed in the intestine. In experimental model of hypercreatininaemia in mouse, montmorillonite is reported to decrease serum creatinine by absorbing that in GI tract and accelerating its excretion from the intestine (44). Urea is the primary metabolite derived from dietary protein and tissue protein turnover. As kidney function decreases, the BUN (blood urea nitrogen) level rises. Bentonite is shown to promote the diffusion of urea from blood vessel to intestine, and inhibits the absorption of urea in intestine (45). Then it sounds that bentonite might be beneficial in renal health.

### Antibacterial effects

The number of antibiotic-resistant pathogenic bacteria has substantially increased nowadays. This alarming trend implies the need to identify and assess new antibacterial agents. It is reported that natural geological minerals hold antibacterial properties, which might provide the hope for novel therapeutic compounds (46). It was shown that montmorillonite is able to absorb coliphages T1 and T7 of Escherichia coli in vitro (47). When the clay was mixed with water (2–4 parts water to 1 part clay) and incubated for 24 hours with live bacteria at body temperature (37°C), a broad spectrum of bacteria was killed (46). Some modified menmorillonits have also shown to exert antibacterial effect (48–50). These antibacterial effects might result from physical interaction (i.e. penetration or rupturing of the cell) and/or chemical interaction of the clay with bacteria (i.e. poisoning or nutrient deprivation) (7).

It is shown that bentonite might modulate the immune response of body. Particles of bentonite inhibits lipopolysaccharide- or concanavalin A-induced red blood cell proliferation and antibody response in vitro (51)

### Bone

It is reported that oral bentonite slightly decreases incorporation of calcium in the bones of goats (52). In nutrient-deficient broiler chicks, although bentonite increased food intake, but did not compensate tibia calcium decrement (53).

### Cancer

Montmorillonite has been used as a drug delivery system for drugs used in cancer therapy such as Paclitaxel (54), 5-fluorouracil (55), 6-mercaptopurine (56)

Although a study reported the genotoxic effect of bentonite on human B lymphoblast cells (57), recently bentonite has been shown to inhibit the growth of human cancer cell lines U251 (central nervous system, glioblastoma). It seems that bentonite clay surfaces controls the levels of metabolic growth components (58).

### Other effects

Montmorillonite is reported not only to adsorb thyroxin and triiodothyronine in vitro but also reduces these hormones in hyperthyroidism model mice. It prolongs the sleep time, improves the hypoxia tolerance capacity and reduces the spontaneous activities of those mice (59)

Bentonite is shown to decrease the bleeding and clotting time and therefore is suggested as a haemostatic agent (60, 61).

It is also reported to act as a promising nanoscale size filler in food packaging industry (62)

Indoor volatile organic compounds (VOCs) have been assumed to affect human health. VOCs can either originate from phenolic and benzene-like compounds in building materials and office furniture or from molds (fungi) growing inside improperly ventilated or sealed buildings. Interestingly it is mentioned that volcanic materials, clays and minerals such as montmorillonite filter VOCs and thereby limit human exposure to these dangerous compounds (63).

### Adverse effects

Besides the above mentioned beneficial effect of bentonite, some unwanted effects are also reported. In vitro studies have shown that bentonite increases cell lysis in some cellular lines while has no effect on some others (64, 65).

A suspected case of bentonite poisoning was reported in a cat which was known to ingest bentonite-containing cat litter. This toxicity led to hypokalemia and hypochromic anemia presented with lethargy and muscle weakness (66). In a 3-year-old girl treated with oral and rectal bentonite as a home remedy, severe hypokalemia occurred which might occur due to the gastrointestinal binding of essential electrolytes (67). Generally it sounds that like any other drug, big doses of bentonite can have some side effects and thereby it is necessary to use a therapeutic dose of this mineral in diseases.

## Conclusion

This article reviewed the papers related to the impact of bentonite on body health. It seems that bentonite, as an abundant natural element, holds the properties to be regarded as a therapy in a broad spectrum of disorders. By searching between more than 2500 articles in Pubmed about bentonite clay, it was determined that about 100 articles were related to the effect of bentonite on body function. The results, which were reviewed in this article, suggest that bentonite has enough encouraging characteristics to merit further investigation. Considering that Mother Nature has a cure for everything, the assessment of natural elements such as this clay should be considered in modern medicine.

## Ethical considerations

Ethical issues (Including plagiarism, informed consent, misconduct, data fabrication and/or falsification, double publication and/or submission, redundancy, etc.) have been completely observed by the authors.
